# Acute Idiopathic Compartment Syndrome of the Forearm in an Adolescent

**DOI:** 10.5811/westjem.2014.9.23019

**Published:** 2014-11-11

**Authors:** Kelley Smith, Robert W. Wolford

**Affiliations:** University of Illinois College of Medicine at Peoria, Department of Emergency Medicine, Peoria, Illinois; OSF Saint Francis Medical Center, Department of Emergency Medicine, Peoria, Illinois

## Abstract

Acute compartment syndrome (ACS) is a condition typically associated with long bone fractures or severe trauma; however, non-traumatic etiologies also occur. We describe a case of an otherwise healthy female pediatric patient presenting with unilateral forearm pain without an inciting injury. Intracompartmental pressures of the forearm were measured and she was diagnosed with idiopathic compartment syndrome. Our goal is to encourage clinicians to consider acute compartment syndrome even in the absence of trauma.

## INTRODUCTION

Acute compartment syndrome (ACS) is an orthopedic emergency that develops when the pressure within a closed fascial space rises and compromises the perfusion to that compartment. It is most commonly seen in the limbs but can occur in other parts of the body. Fractures are the most common etiology of ACS (nearly 75%), and the most common associated with ACS is the tibia.^1^ However, ACS can occur in the absence of fracture or other injury.

## CASE REPORT

A 17-year-old Caucasian female presented to the emergency department with the complaint of left forearm pain. She initially noted paresthesia of the left hand and then left forearm pain. The pain had been progressive over the previous 10 hours and was exacerbated with flexion or extension of the wrist. She could not recall any injury or inciting event. She was otherwise healthy and without any significant past medical history. Her family history was unremarkable with the exception of her mother’s brother who died of Duchenne muscular dystrophy. Her mother had been tested for Duchenne carrier status and was negative. Her only medication was an oral contraceptive. She denied the use of tobacco, alcohol, and illicit drugs. Her initial vital signs were an oral temperature of 36.8°C, pulse of 76 beats per minute, blood pressure of 144/96 mm Hg, respiratory rate of 16 breaths per minute, and room air pulse oximetry of 100%. The physical examination of the left forearm revealed it to be firm and tender to palpation, and it measured 2cm larger in circumference than the right. Her radial pulse was palpable and her fingertip capillary refill was less than two seconds. The skin appeared normal without overlying erythema or calor. The patient experienced pain with flexion or extension of the digits and wrist. No sensory deficits of the hand or forearm were noted. Her initial laboratory evaluation showed the following: leukocytosis of 15,300 10(3)/mcL, blood glucose of 93mg/dl, elevated creatine phosphokinase of 15, 750U/L, normal erythrocyte sedimentation rate, and slightly elevated c-reactive protein at 1.27mg/L. No coagulation studies were obtained. A radiograph of the forearm was obtained and demonstrated only soft tissue swelling. Venous and arterial duplex studies of the extremity were performed. The arterial duplex demonstrated no evidence of arterial thrombosis, stenosis, or occlusion. The venous studied showed normal flow and compressibility, but the waveforms were continuous. Compartment pressures were measured in the volar and dorsal compartments of the forearm with a Stryker Needle™ (Stryker Intra-Compartmental Pressure Monitor, Stryker Corporation, USA) and found to be 86mmHg and 62mmHg, respectively. The patient was taken to the operating suite and underwent an emergent 10cm long fasciotomy of both the volar and dorsal compartments of her left upper extremity. Intraoperative cultures were obtained and showed no growth. Fortunately, the patient required minimal debridement. Total hospital course was four days. A precipitating cause could not be identified. The patient’s fasciotomy sites were surgically closed and she was discharged home with a diagnosis of idiopathic compartment syndrome. At 18-month follow up, the etiology of the compartment syndrome has not been identified, although she has been found to have a chronically elevated creatine phosphokinase (1,500U/L).

## DISCUSSION

As emergency physicians, we must be vigilant and consider ACS. ACS occurs in a variety of clinical situations and in various anatomic locations. It is most commonly associated with a fracture or other traumatic injury, such as a crushing mechanism. It can also develop in the setting of hereditary bleeding disorders, therapeutic anticoagulation, septicemia, animal bites, or venous cannulation.^2^ As our case demonstrates, it may even be idiopathic (Table).

ACS was first described in 1881, when Richard von Volkmann defined the contracture that is commonly seen as a complication.^3^ About 1%–10% of patients with ACS may go on to develop Volkmann’s ischemic contracture.^4^ ACS develops whenever the pressure inside the fascial compartment increases, whether that is caused from external compression, internal edema, or reperfusion. As the pressure rises, venous outflow is compromised first, followed eventually by arterial flow. Ischemia to the nerves and muscle develops. Vasodilatation is a compensatory mechanism to increase perfusion, but eventually leads to increased vascular permeability. The lymphatic system is overwhelmed and the compartmental pressure increases, resulting in decreased perfusion and eventually necrosis.

In most cases, ACS develops in the setting of a closed fracture. In a systematic review by Kalyani et al.,^5^ the most common cause of ACS in the upper extremity in adults is a fracture of the distal radius; however, in pediatrics the most common etiology is a supracondylar fracture of the humerus. Other locations for ACS include the hand, forearm, arm, shoulder, back, buttocks, thigh, and foot.^1^ It should also be noted that 23% to 31% of ACS cases occur in the setting of soft tissue injuries without the presence of a fracture.^2^,^5^–^7^

Classically, ACS is associated with “The 5 Ps:” Pain, pallor, paresis, paresthesias, and pulselessness. Of these signs, pain out of proportion to the injury is the first to develop and is more commonly seen; however, the loss of normal neurologic sensation is the most reliable sign.^1^ In one study of 90 patients by Duckworth et al.,^6^ edema was noted in 100% of cases, followed next by pain in 79%, and then by paresthesias in 52% of patients. In the case of our patient, she had swelling along with pain with passive extension or flexion of the wrist. She also had a very tense and tender forearm to palpation. A pediatric study by Bae et al.,^8^ found that 90% of patients reported pain. Seventy percent had pain associated with one other “P,” leaving 30% of patients with pain as the only symptom. They suggested close monitoring of the analgesic requirement by the child, as this could serve as an additional indicator of the development of ACS. They found that the need for increased pain medication preceded other clinical symptoms of ACS by an average of 7.3 hours in a sub-set of patients who had access to patient-controlled analgesia. Gelberman et al.,^9^ found that diminished two-point discrimination was a reliable finding and was sensitive in differentiating ACS from a less acute injury.

Compartment pressures should be measured whenever ACS is considered. ACS in the setting of soft tissue injury alone is more likely to result in delay of treatment and the development of complications. One study found that ACS patients without a fracture had an average delay to fasciotomy that was longer by 12.4 hours.^7^ Our patient’s compartment pressures were measured using the Stryker Needle™. Normal pressure is usually considered to be <10mmHg. Patients typically notice pain as pressures increase to 20–30mmHg.^3^ Capillary blood flow is compromised as pressures approach 25–30mmHg. Mubarak et al.,^10^–^12^ recommend using an intracompartmental pressure (ICP) of more than 30mmHg as an indication for performing fasciotomy, stating that once the critical ICP of 30 mmHg is reached, permanent damage can occur in as little as 6–8 hours. Management of ACS focuses on the maintenance of adequate perfusion pressure (diastolic blood pressure minus the ICP). A pressure difference less than 30mmHg for more than two hours is considered diagnostic of a compartment syndrome.^6^ Hypotension must be prevented in patients with ACS. Once ACS is diagnosed, any source of external compression, such as a splint or cast, should be removed immediately. If a cast is bivalved, the compartment pressures may decrease as much as 55%, and if the cast is entirely removed, the pressure may be reduced as much as 85%.^1^ The limb should be placed at the level of the heart and adequate analgesia and supplemental oxygen provided as needed. ICP should be measured in all compartments of the extremity. Other complications of ACS, including rhabdomyolysis, hyperkalemia, and myoglobinuria should be sought.

In summary, our patient had acute compartment syndrome of the forearm without a history of trauma or other identifiable cause. Other reports of “idiopathic” ACS have involved adults and the leg.^13^–^15^ A retrospective study of pediatric patients, with upper extremity compartment syndrome without a fracture, cared for at a large academic center over a 22-year period, found the majority to be iatrogenic (ex. infiltrated intravenous line) or to be associated with an infection.^16^ None were “idiopathic,” as in this case.

Acute compartment syndrome is frequently not considered in patients without a fracture or other traumatic injury. ACS is a clinical diagnosis. Care providers must have a high clinical suspicion for compartment syndrome in patients without injuries but with pain out of proportion to clinical findings, as demonstrated by our patient, to prevent irreversible damage.

## Figures and Tables

**Table:** Etiology of acute compartment syndrome.

External compression	Internal compression
Circumferential cast or dressing	Hematoma/coagulopathy
Burn eschar	Fracture
Military anti-shock trousers (MAST)	Overexertion of muscles (seizure)
Compression from prolonged immobility	Post-ischemic time/reperfusion
	Animal bite/envenomation
	Soft tissue injury
	Vascular injury
	Deep venous thrombosis
	Idiopathic
